# Azygos vein approach for radiofrequency ablation of para-Hisian atrial tachycardia in a patient with inferior vena cava interruption

**DOI:** 10.1016/j.hrcr.2025.01.009

**Published:** 2025-01-24

**Authors:** Atsuhito Oda, Yuichiro Sagawa, Yumi Yasui, Hirofumi Arai, Kazuya Murata, Kaoru Okishige, Manabu Kurabayashi, Yasuteru Yamauchi

**Affiliations:** Heart Center, Japan Red Cross Yokohama City Bay Hospital, Yokohama City, Japan

**Keywords:** Azygous vein, Radiofrequency ablation, Para-Hisian atrial tachycardia, Interruption, Inferior vena cava

**Key Teaching Points**

• The azygos vein approach offers a feasible and effective alternative for radiofrequency ablation in patients with an interrupted inferior vena cava, addressing challenges in conventional femoral access.
• The azygos vein approach using long sheaths allows the catheter tip to parallel contact with the tissue near the His-bundle, and the catheter is aligned along the greater curvature of the azygos vein, thereby facilitating precise and stable catheter positioning.
• Detailed electroanatomic mapping is crucial for safe and effective ablation near the atrioventricular node to ensure accurate identification of the source of tachycardia and minimize the risk of atrioventricular conduction disturbances.

## Introduction

Infrahepatic interruption of the inferior vena cava (IVC) is a congenital anomaly found in 0.15% of the general population and in 0.6% of patients with congenital heart disease.^1^^,^^2^ Because of its asymptomatic nature and lack of functional limitations, this anomaly is often detected incidentally during cardiac intervention. Catheter ablation is widely performed using a trans-IVC approach in the right heart. In cases of IVC interruption, alternative approaches, such as the internal jugular, subclavian, and azygos vein approaches, are used. Here, we report the first case of successful ablation of para-Hisian atrial tachycardia (AT) using the azygos vein approach in a patient with an interrupted IVC.

## Case Report

A 73-year-old woman presented with recurrent palpitations. Holter electrocardiography revealed a narrow QRS tachycardia characterized by sudden onset and termination. Transthoracic echocardiography showed normal left ventricular function and could not reveal any structural abnormalities. With informed consent, electrophysiological study and catheter ablation were performed.

**Figure 1:**
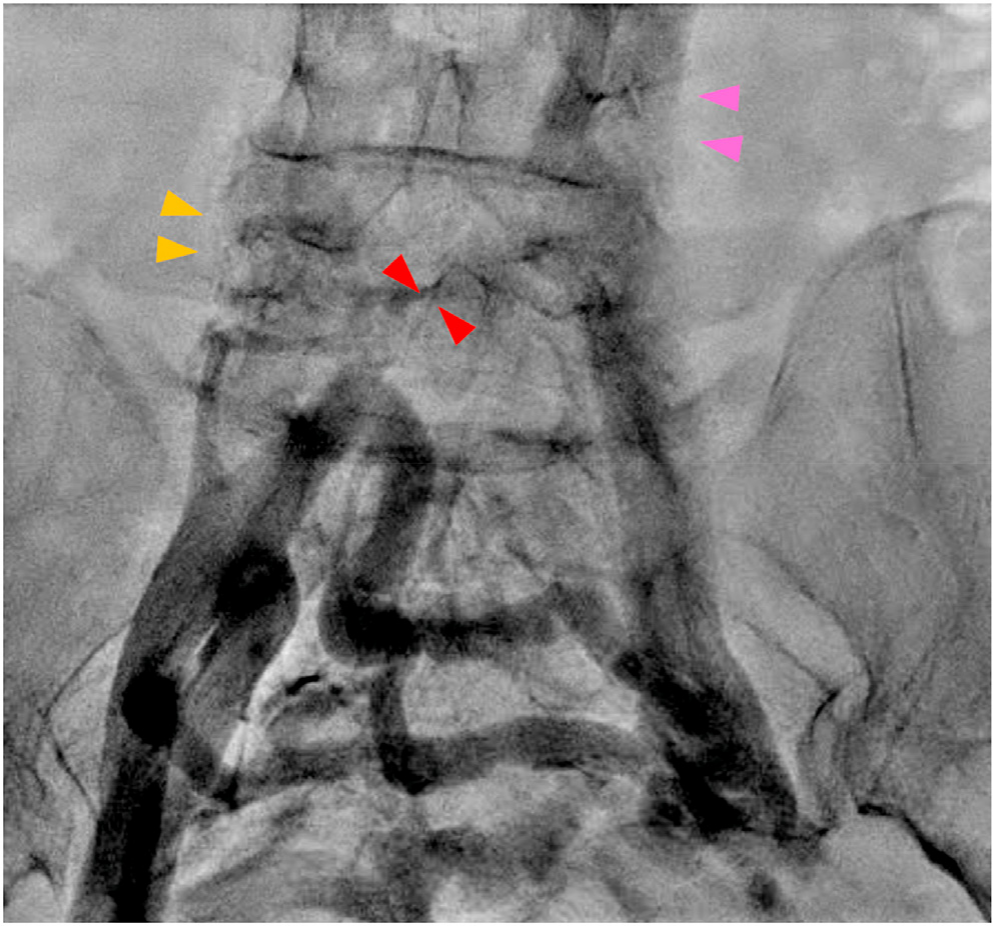
Venography is performed by inserting a sheath into the right femoral vein. The inferior vena cava is absent (*yellow arrowheads*), the right common iliac vein is narrowed (*red arrowheads*), and collateral circulation begins with visualization of the left common femoral vein. The left common femoral vein is connected to the infrarenal inferior vena cava via the hemiazygos vein (*pink arrowheads*), which develops on the left side of the vertebrae.

The initial access to the right atrium (RA) via the right femoral vein showed an unusual catheter course. Venography indicated an interruption of the IVC with a dilated azygos vein draining into the superior vena cava (SVC) (Figure 1). Severe stenosis was observed at the confluence of the left common iliac and azygos veins. We i]nserted a catheter sheath through the left femoral vein and accessed the RA via the SVC through the azygos vein.

AT was induced by programmed stimulation of the RA, presenting as a long RP narrow complex tachycardia at 130 beats/min with 1:1 atrioventricular (AV) conduction. Ventricular overdrive pacing displayed a V-A-A-V response, whereas differential atrial overdrive pacing showed no VA linking. Activation mapping with a high-density grid multipolar catheter (HDG; Abbott Technologies, Minneapolis, MN) guided by a 3-dimensional (3D) electroanatomic mapping system (Ensite NavX, Abbott Technologies) identified focal AT with the earliest activation in the para-Hisian region. Administration of 2 mg adenosine triphosphate intravenously terminated the AT without causing an AV block, suggesting that focal AT originated near the His bundle region with high adenosine triphosphate sensitivity.

Radiofrequency (RF) ablation was initially attempted in the noncoronary cusp (NCC) using a transaortic approach to avoid damaging the AV node. The atrial potential identified as the earliest activation site within the NCC preceded the onset of the body surface P-wave by 15 ms and was comparable in timing to the atrial potential recorded at the coronary sinus (CS) ostium, indicating similar early activation. RF energy was initially applied at the earliest activation site within the NCC at 30 W and then increased to 35 W. Despite AT termination after 4.5 seconds of RF application, AT was repeatedly induced even after multiple additional RF applications in the NCC.

**Figure 2:**
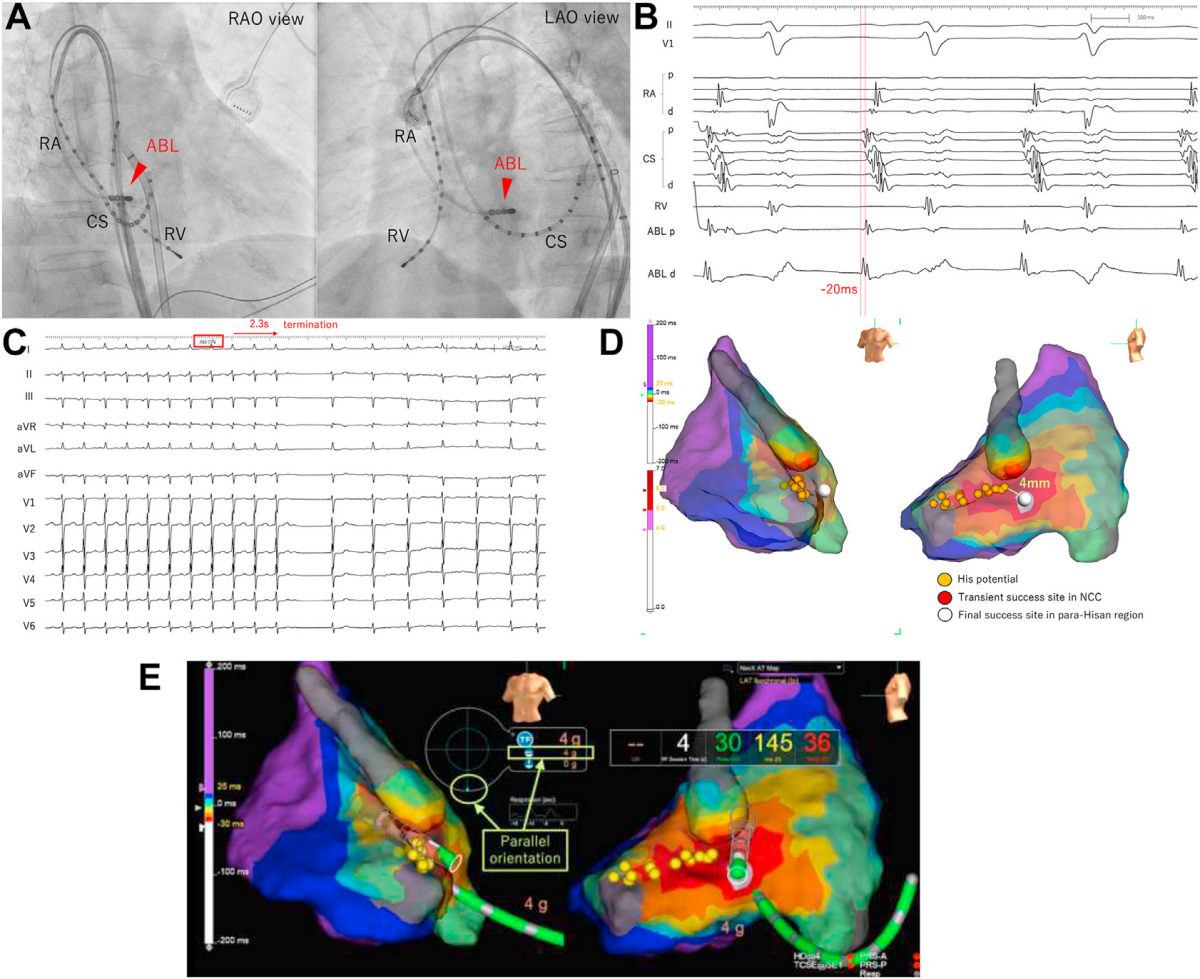
**A:** An irrigated ablation catheter is advanced through the steerable sheath and located at the earliest activation site in the para-Hisian region. ABL = ablation catheter; CS = coronary sinus; LAO = left anterior oblique; RA = right atrium; RAO = right anterior oblique; RV = right ventricle. **B:** The earliest activation site is located in the para-His region, where atrial signals are detected 20 ms pre-QRS. No His potentials are recorded on the ablation catheter. **C:** Atrial tachycardia is terminated 2.3 seconds after radiofrequency ablation. ABL = ablation catheter; CS = coronary sinus; RA = right atrium; RV = right ventricle. **D:** The activation map during atrial tachycardia shows the locations where ablation is performed and His potentials are recorded. The *red tag* indicates the transiently successful ablation sites from the noncoronary cusp, and the *white tag* indicates the final successful ablation sites in the para-Hisian area. LAO = left anterior oblique; LL = left lateral; NCC = non-coronary cusp. **E:** The contact force data at the final successful ablation site show that the vector of contact between the catheter tip and tissue lacks any perpendicular component, consisting entirely of parallel vectors. LAO = left anterior oblique; LL = left lateral.

**Figure 3:**
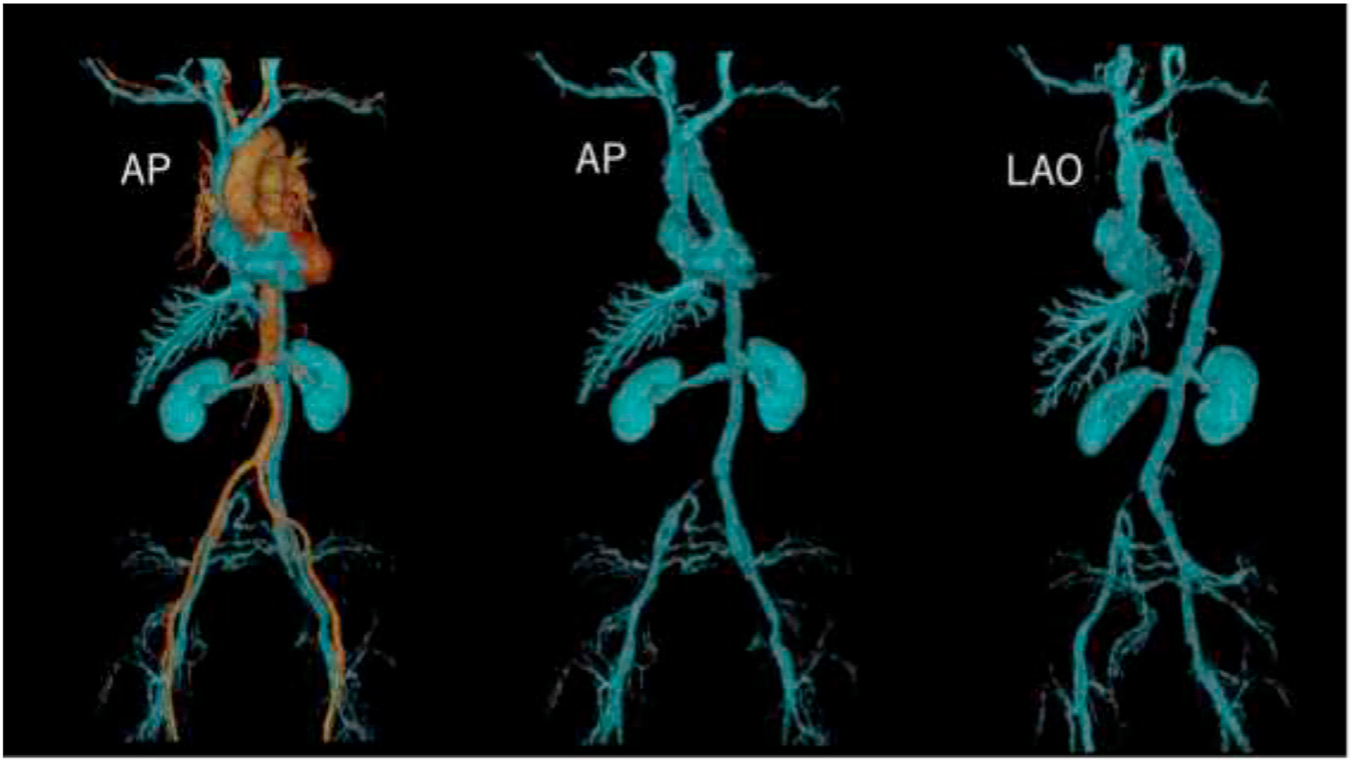
A postoperative 3D computed tomography scan shows that the infrahepatic segment of the inferior vena cava (IVC) is absent, and the infrarenal IVC begins with a developed azygos vein system, perfusing from the superior vena cava (SVC) to the right atrium. The hepatic veins are draining into the right atrium via the remaining thoracic IVC. The right common iliac vein is narrowed because of compression by the left common iliac artery. 3D = 3-dimensional; AP = anteroposterior; LAO = left anterior oblique.

Subsequently, a trans-SVC approach via the azygos vein was used through a deflectable sheath (Agilis, Abbott Technologies). The earliest atrial activation site in the RA was located near the His bundle region, where the atrial potentials at the ablation catheter were recorded 20 milliseconds before the onset of the body surface P-wave, slightly preceding the atrial potential recorded at the CS ostium. (Figure 2A, 2B). RF energy was initially applied at 20 W, resulting in AT termination within 2.3 seconds (Figure 2C). The RF delivery was continued for 60 seconds, with the power gradually increasing to 30 W. The successful ablation site, as measured using the 3D mapping system, was 4 mm from the His-potential recording site (Figure 2D). The tip of the catheter was in parallel contact with the tissue, providing stability (Figure 2E; Supplemental Video). No AV conduction disturbances were observed during or after ablation. AT was not induced by atrial burst pacing during isoproterenol infusion. Postoperative computed tomography confirmed the absence of other anomalies (Figure 3).

## Discussion

AT originating near the AV node accounts for approximately 6% of focal AT cases.^3^ Para-Hisian AT typically exhibits electrophysiologic characteristics that respond intravenously to a small amount of adenosine, such as 5 mg or less, a feature that was also confirmed in this case.^4^

Radiofrequency ablation near the AV node has been reported to be highly effective; however, it naturally carries the risk of AV conduction disturbances. Successful cases of NCC ablation have been reported, in which the earliest atrial activation site in the NCC was identified.^5^ In these instances, the activation in the NCC occurred earlier than that in the right or left atrium. In contrast, in this case, the RA exhibited slightly earlier activation than the NCC, which may account for the transient success observed in NCC ablation.

It is crucial to perform detailed mapping of the AT near the AV node, including the NCC, during ablation. This approach helps minimize risks to the AV conduction system and aids in accurately identifying the source of tachycardia. Such meticulous mapping ensures precise targeting, thereby enhancing the safety and efficacy of ablation treatment.

The azygos vein approach was necessitated by IVC interruption, which complicates conventional femoral access typically used in electrophysiological procedures. Previous reports have documented the use of the azygos vein approach for other types of arrhythmias, such as AV nodal reentrant tachycardia (AVNRT), posteroseptal/anteroseptal accessory pathways, and typical atrial fiutter,^6^, ^7^, ^8^, ^9^, ^10^, ^11^, ^12^ but not specifically for para-Hisian AT. To our knowledge, this is the first case report on the use of the azygos vein approach for the ablation of para-Hisian AT. Ablation near the His bundle carries the risk of an AV block, making catheter stability crucial.

The efficacy of ablating the para-Hisian accessory pathways using the SVC approach from the internal jugular vein also has been reported^13^^,^^14^ In the conventional IVC approach, the catheter contacts the tissue perpendicularly, whereas in the SVC approach, the catheter tip is anchored to the annulus, making contact with the tissue in a parallel orientation, thereby improving stability. Enhanced catheter stabilization leads to higher acute success rates and lower recurrence rates.^14^ In this case, the tip of the ablation catheter was fixed for the same reasons. Additionally, the stability was further improved by aligning the catheter along the greater curvature of the azygos vein, ensuring precise delivery of RF energy and minimizing the risk of complications. The azygos vein approach, reported to be challenging because of the longer course and sharp angulation at the entry point of these venous channels into the SVC, can benefit from the use of long sheaths, which enhance catheter stability.^15^ In this case, we used long sheaths and successfully terminated the tachycardia. The maintenance of sinus rhythm after ablation indicates the efficacy of this approach.

Cryoablation has been reported to carry a lower risk of AV conduction block compared with RF ablation in the treatment of AVNRT. Although the acute success rates are comparable between the 2 modalities, cryoablation has been associated with higher rates of recurrence in the chronic phase.^15^ Cryoablation catheters lack contact force sensing, which poses challenges in cases involving complex anatomy, as demonstrated in the current case. When employing an atypical approach (with the catheter traversing a long intravascular path and multiple bends), the absence of contact force guidance may result in insufficient catheter contact for effective ablation. In this case, we considered switching to a cryoablation catheter if stable contact with the RF catheter could not be achieved, because unstable positioning of the RF catheter might increase the risk of AV conduction block. However, we ultimately succeeded in achieving stable contact with the RF catheter.

A systematic review of electrophysiological procedures in 34 patients with IVC obstruction who underwent ablation for AVNRT or AV nodes was reported. Among these cases, 20 used the superior approach (via the right internal jugular vein or the right subclavian vein), 11 used the inferior approach via the azygos vein (2 with the retrograde transaortic approach and 1 with the transhepatic approach).^16^ This superior approach has the advantage of being relatively straightforward to perform; however, it has the disadvantage of increasing radiation exposure to the operator. The transhepatic approach carries the risk of complications such as bleeding, infection, hepatitis, pancreatitis, pneumothorax, gallbladder perforation, and hepatic vein thrombosis, and the limited experience with this approach is also a drawback. The inferior approach overcomes these disadvantages, while presenting challenges to catheter manipulation.

## Conclusion

This case highlights the successful use of the azygos vein approach for RF ablation of para-Hisian AT in a patient with an interrupted IVC. Detailed electroanatomic mapping, parallel catheter tip contact, and the use of long sheaths facilitated precise catheter positioning, enabling effective ablation and minimizing the risk of complications. This approach provides a viable alternative to catheter ablation for patients with complex venous anomalies.

## Disclosures

The authors declare no conflicts of interest.
