# Supplementary material for: Azygos vein approach for radiofrequency ablation of para-Hisian atrial tachycardia in a patient with inferior vena cava interruption
Source: HeartRhythm Case Rep. 2025 Jan 24;11(4):343–6. doi: 10.1016/j.hrcr.2025.01.009 (PMC12138071; doi:10.1016/j.hrcr.2025.01.009)
Supplement: Supplemental Video Legend [file mmc2.docx]

**Supplemental Video**

This video shows good catheter stability at the final successful ablation site. Tachycardia is terminated 2.3 s after ablation. Ablation is continued while confirming the absence of an atrioventricular conduction block during atrial burst pacing.
